# Pre-existing minor variants with NS5A L31M/V-Y93H double substitution are closely linked to virologic failure with asunaprevir plus daclatasvir treatment for genotype 1b hepatitis C virus infection

**DOI:** 10.1371/journal.pone.0234811

**Published:** 2020-06-16

**Authors:** Naoki Morishita, Ryotaro Sakamori, Tomomi Yamada, Yugo Kai, Yuki Tahata, Ayako Urabe, Ryoko Yamada, Takahiro Kodama, Hayato Hikita, Yoshinori Doi, Shinji Tamura, Hideki Hagiwara, Yasuharu Imai, Sadaharu Iio, Tomohide Tatsumi, Tetsuo Takehara

**Affiliations:** 1 Department of Gastroenterology and Hepatology, Graduate School of Medicine, Osaka University, Suita, Osaka, Japan; 2 Department of Medical Innovation, Osaka University Hospital, Suita, Osaka, Japan; 3 KKR Otemae Hospital, Osaka, Osaka, Japan; 4 Minoh City Hospital, Minoh, Osaka, Japan; 5 Kansai Rosai Hospital, Amagasaki, Hyogo, Japan; 6 Ikeda Municipal Hospital, Ikeda, Osaka, Japan; 7 Hyogo Prefectural Nishinomiya Hospital, Nishinomiya, Hyogo, Japan; University of Cincinnati College of Medicine, UNITED STATES

## Abstract

**Background:**

L31 and Y93 in the NS5A region of the hepatitis C virus (HCV) are the most important substitution positions associated with resistance to direct-acting antiviral (DAA) treatment.

**Methods:**

We analyzed the frequency of NS5A L31M/V and Y93/H in NS5A inhibitor-naive HCV genotype 1 patients who received asunaprevir plus daclatasvir combination treatment using a conventional sequencing method and a deep sequencing method that can distinguish a single substitution at either position and a double substitution at both positions with a 0.1% detection threshold.

**Results:**

The frequency of substitutions at both sites using the conventional method was very low, with 1 in 14 non-responders and 0 in 42 randomly selected responder patients. On the other hand, for the deep sequencing method, cases with double substitutions in the tandem sequence were detected in 8/14 non-responders and 1/42 responders (p<0.0001). For the conventional method, substitutions were detected at any position in 6/14 non-responders and 2/42 responders (p = 0.0019), with a clear difference between the two groups. The difference was also clear with the deep sequencing method, with 11/14 non-responders and 8/42 responders. Interestingly, for the deep sequencing method, the single substitution of L31 was found in 6/14 non-responders and 7/42 responders, whereas single substitutions of Y93 or double substitutions were found in 7/14 vs. 1/42 and 8/14 vs. 1/42 patients, respectively.

**Conclusions:**

NS5A L31 and Y93 substitutions were detected in tandem by the deep sequencing methods in several genotype 1 patients, who may be more resistant to DAA treatment containing an NS5A inhibitor.

## Introduction

Chronic hepatitis C virus (HCV) infection affects an estimated 71 million people worldwide and results in liver cirrhosis and hepatocellular carcinoma [1]. The therapeutic efficacy for HCV infection remarkably improved along with a paradigm shift from interferon (IFN)-containing therapy to IFN-free therapy with direct-acting antiviral agents (DAAs). HCV genotype 1 predominates in the USA, Europe and Asian Pacific and has been difficult to treat [2]. Genotype 1a is most common in the USA [3], and genotype 1b is most common in Eastern Europe, Latin America and Eastern Asia [4].

The combination of the NS3 protease inhibitor asunaprevir (ASV) and the pan-genotypic NS5A inhibitor daclatasvir (DCV) demonstrated poor response for genotype 1a in a phase 2a trial [5], whereas high virologic response for genotype 1b in Japanese and multinational phase 3 trials [6, 7] was approved in many countries where genotype 1b is common. However, an Invader assay in a clinical trial [6, 7] indicated that therapeutic efficacy was decreased when resistance-associated pre-existing substitutions (RASs) of L31M/V and/or Y93H in the NS5A region were present. Furthermore, the emergence of L31 and/or Y93 substitutions was frequently observed by an Invader assay after virologic failure and persisted for a long period thereafter [8, 9]. Thus, L31 and Y93 in the NS5A region are the two most important RASs associated with virologic failure of ASV/DCV therapy, and investigations of the presence of NS5A RASs before initiation of NS5A inhibitor-containing regimens are important for tailoring the treatment.

Thus, pre-existing linked L31M/V-Y93H double substitutions could affect the virologic response of ASV/DCV therapy, even though their prevalence is low. Moreover, RASs in the NS5A sequence also affect other DAA treatments with ledipasvir or ombitasvir [10]. However, conventional sequencing methods cannot detect linked L31M/V-Y93H double substitutions in a single clone. Recently, deep sequencing was used to analyze RASs and has some advantages for the detection of HCV quasispecies dynamics [11]. The development of deep sequencing technology enabled us to establish methods for detecting linked L31M/V-Y93H double substitutions without fragmentation. Using this novel deep sequencing method and phylogenetic tree analysis, we reported that pre-existing minor L31M/V-Y93H double-substituted variants could occasionally contribute to double substitutions after ASV/DCV failure [12]. However, the significance of these substitutions on treatment outcome, let alone their prevalence, remains unknown. Thus, we conducted a prospective multicenter study of chronic HCV genotype 1 infection treated with an NS5A inhibitor-containing regimen and investigated the impact of pre-existing L31M/V-Y93H double substitutions on treatment outcome using a novel deep sequencing method in this study.

## Methods

### Patients

**Fig 1:**
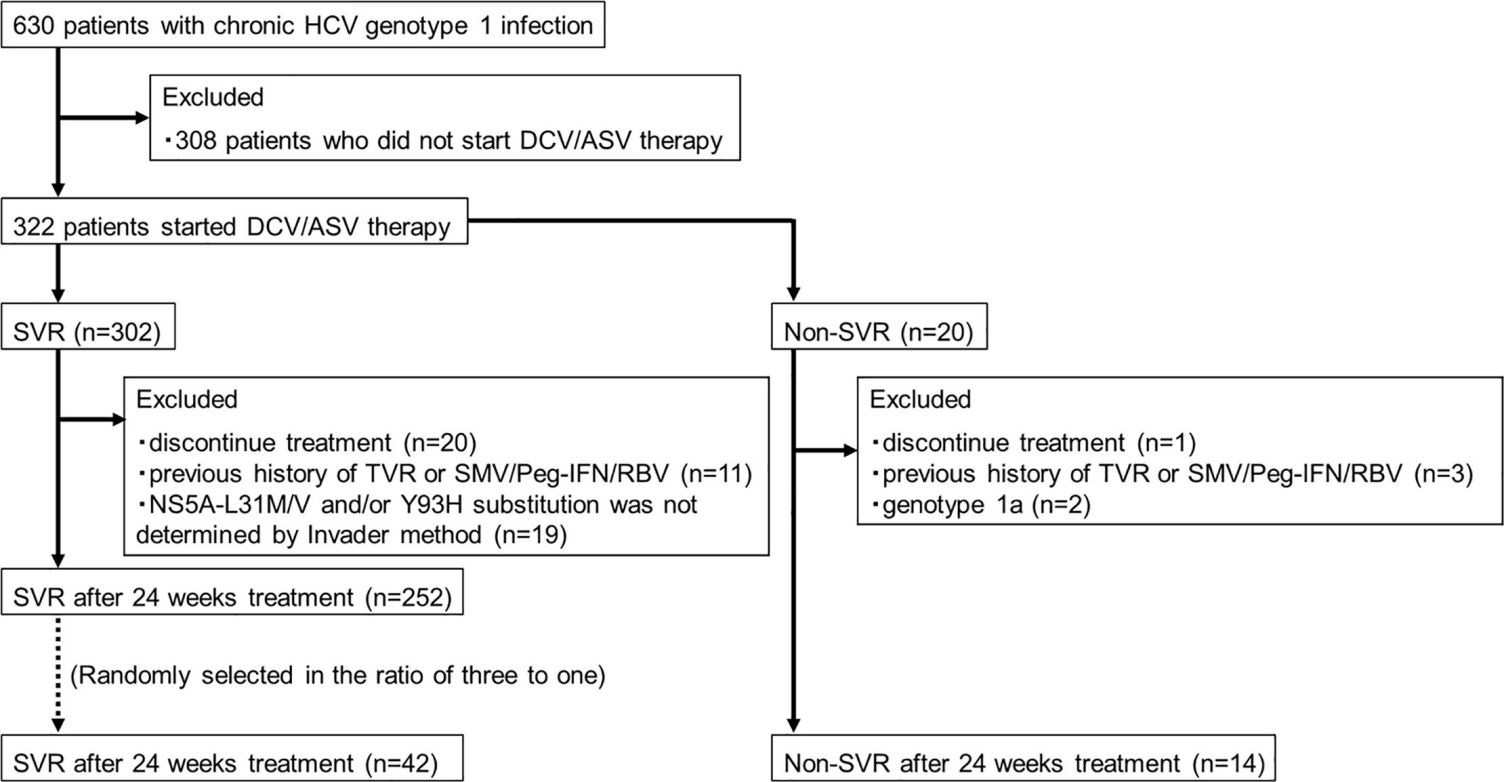
Flow chart of all enrolled patients.

The present study was performed using data and blood samples from a prospective, multicenter study conducted by Osaka University Hospital and 21 other institutions participating in the Osaka Liver Forum. Overall, 630 consecutive patients with chronic HCV genotype 1 infection were enrolled in this study (**Fig 1**). Prior to initiation of ASV/DCV therapy, a drug-resistant test using the Invader assay was performed to investigate the presence of RASs in NS5A regions and to assess the indication for treatment. After evaluating the results of the Invader assay, 308 patients did not start ASV/DCV therapy due to inconvenience or because they were awaiting other DAA therapies. As a result, the remaining 322 patients started ASV/DCV therapy for 24 weeks between September 2014 and August 2015. ASV was administered orally at a dose of 100 mg twice a day, and DCV was administered orally at a dose of 60 mg once a day (Bristol-Myers Squibb, Tokyo, Japan). Eligible patients were aged 20 years or older with chronic HCV genotype 1 infection. Patients with hepatocellular carcinoma, hepatitis B or human immunodeficiency virus coinfection, Child-Pugh B or C cirrhosis and decompensated cirrhosis, and other forms of liver disease (e.g., autoimmune hepatitis or alcoholic liver disease) were excluded from this study. This study was approved by the institutional review boards of each participating institution (Osaka University Hospital, KKR Otemae Hospital, Minoh City Hospital, Kansai Rosai Hospital, Ikeda Municipal Hospital, Hyogo Prefectural Nishinomiya Hospital, Osaka International Cancer Institute, Osaka Rosai Hospital, Osaka Police Hospital, Japan Community Healthcare Organization Osaka Hospital, Osaka General Medical Center, Toyonaka Municipal Hospital, Suita Municipal Hospital, Yao Municipal Hospital, Itami City Hospital, Higashiosaka City Medical Center, Nishinomiya Municipal Central Hospital, Kaizuka City Hospital, Sumitomo Hospital, Saso Hospital, and NTT West Osaka Hospital) and is registered with University Hospital Medical Information Network (UMIN) 000015247) in accordance with the ethical guidelines of the 1975 Declaration of Helsinki amended in 2008. Written informed consent was obtained from all patients before participating in the study.

### Assessment of clinical, laboratory, and liver histology results and the virologic response

Clinical data were measured at baseline, several points during treatment and 4, 12 and 24 weeks after the end of treatment. Blood samples were obtained at baseline, day 2, weeks 1, 2, 4, 12 and 24 during treatment and 4, 12 and 24 weeks after the end of treatment. Liver biopsy was performed before initiation of ASV/DCV therapy, and the grade of activity and the stage of fibrosis were evaluated in reference to the METAVIR score. HCV-RNA levels were measured by the COBAS Taqman HCV Test, version 2.0 (lower limit of detection 1.2 log IU/ml; Roche Diagnostics, Branchburg, NJ, USA). A sustained virologic response was defined as an undetectable serum HCV-RNA level 24 weeks after the end of treatment.

### Invader assay

RAS detection in the NS5A region at baseline was performed by Invader assay (BML, Inc., Saitama, Japan) [13, 14]. The targeted mutations included L31F/M/V and Y93H in the NS5A region. The Invader assay has been used for high-throughput single nucleotide polymorphism (SNP) genotyping [15], and because of its accuracy and quantitative nature, the Invader assay has also been used for analysis of mRNA [16]. This method was divided into two reactions. First, invader oligonucleotide and allele-specific probes anneal with the target to form one base overlap. When the base is complementary to the opposing base in the allele-specific probe, cleavage recognizes the structure and releases the 5’ flap. The released flap anneals to a fluorescence resonance energy transfer (FRET) probe. The second cleavage reaction releases fluorophore, resulting in the generation of a fluorescent signal, the strength of which is determined quantitatively [17]. In this study, we used a commercially available Invader assay for detection of RASs in the NS5A region at baseline (BML, Inc., Saitama, Japan) [14, 18]. The targeted mutations were L31F/M/V and Y93H in the NS5A region. The results of RASs were determined to be negative (<1%), weak positive (1–20%) and positive (>20%) according to the signal strength of the Invader reaction [19]. We defined positive as significant in this study. The results for RASs were determined to be negative (<1%), weakly positive (1–20%) and positive (>20%) according to the signal strength of the Invader reaction [18]. We defined a positive result as significant in this study.

### Deep sequencing analysis

Deep sequencing was performed to detect RASs in the NS5A region using an Ion Personal Genome Machine (PGM) Sequencer (Thermo Fisher Scientific Inc., MA, USA). First, HCV-RNA was extracted from 140 μl of serum samples using the QIAamp® viral RNA Mini Kit (QIAGEN, Hilden, Germany), and the extracted RNA was reverse transcribed with genotype 1 HCV-specific primer using SuperScript III Reverse Transcriptase (Thermo Fisher Scientific Inc., MA, USA). Then, amplification of the targeted NS5A region was performed by nested PCR and a fusion method without fragmentation according to the instruction manual of Ion Amplicon Library Preparation. The primer sequences were previously described [19]. The first amplification was performed using KOD-Plus-Neo DNA polymerase (Toyobo, Osaka, Japan) with outer primer pairs. To analyze linked L31-Y93 substitutions in a single clone, we designed an inner primer set between which both L31 and Y93 were located. The second amplification was performed with fusion primers containing the inner primer attached by the PGM sequencing adaptor with barcode sequencing using Platinum PCR SuperMix High Fidelity (Thermo Fisher Scientific Inc., MA, USA) called Fusion methods. The amplicon libraries were subsequently purified with Agencourt AMPure XP Reagent (Beckman Coulter, CA, USA). The quantification and size distributions of each amplicon were analyzed by qPCR and the Agilent Bioanalyzer 2100 instrument. The coverage of each sequencing run was at least more than 5000 reads. The results of deep sequencing were analyzed to identify the linked NS5A-L31-Y93 substituted combination in a single amplicon and the frequency of the L31M/V-Y93 single substituted strain, the L31-Y93H single substituted strain and the linked L31M/V-Y93H double substituted strain. To validate the error rates in sequencing approaches, we conducted control experiments using a plasmid encoding wild-type HCV sequence. As previously described, cut-off values were calculated as the mean error rates plus 2 standard deviations of three sequencing runs and resulted in 0.20%, 0.22% and 0.10% for L31M/V-Y93 single substitution, L31-Y93H single substitution and L31M/V-Y93H double substitution, respectively [12].

### Case-control study

To assess the impact of pre-existing L31M/V-Y93H minor clones on the treatment effects of ASV/DCV therapy, we conducted a case-control study with 14 non-SVR cases and 42 SVR controls and compared the predictive capability for the treatment effect between substitution at either position before treatment by conventional Invader methods and double substitutions at these sites by deep sequencing. Patients in the control group were randomly selected from SVR patients in the ratio of three to one for non-SVR patients using the random function in Microsoft Excel.

### Statistical analysis

The baseline quantitative demographic data are expressed as the median and interquartile range. The logistic regression analysis was performed to analyze the association between the baseline data and SVR. All continuous variables were dichotomized according to the median value in the analyses of factors associated with SVR. To analyze the differences between the two groups, Pearson's chi-squared test, Fisher’s exact test, t tests or the Mann-Whitney U test was performed. The differences in sensitivity and specificity between the two groups were analyzed by McNemar’s test. A p-value < 0.05 was regarded as significant for each statistical test. The statistical analyses were performed using SPSS version 23 (SPSS, Chicago, IL, USA).

## Results

### Baseline characteristics of the patients who began ASV/DCV therapy

The characteristics of 322 patients who began ASV/DCV therapy are presented in **Table 1**. The mean age of the patients was 69.5 years. The percentage of cirrhosis was 39.4%. The percentage of patients with a history of previous IFN, IFN/RBV, and Peg-IFN/RBV therapy was 50.3%. Fourteen patients had a history of previous telaprevir (TVR)-based and/or simeprevir (SMV)-based triple therapy. The numbers of patients positive for NS5A-L31M/V and NS5A-Y93H by Invader assay were 5 (1.7%) and 10 (3.3%), respectively. Only one patient was positive for both NS5A-Y93H and NS5A-L31M/V.

**Table 1:** Baseline characteristics of patients who received DCV/ASV therapy.

Factor	n = 322
Age (y.o)	71 (65–76)
Sex: male/female	144 / 178
Body mass index (kg/m^2^)	22.6 (20.7–24.8)
Chronic hepatitis /Liver cirhhosis	195 / 127
Previous IFN, IFN/RBV, Peg-IFN/RBV	162
Previous TVR/Peg-IFN/RBV	6
Previous SMV/Peg-IFN/RBV	10
HCV-RNA (Log IU/ml)	6.1 (5.7–6.4)
Liver histology Fibrosis: F0/1/2/3/4	12 / 54 / 52 / 34 / 35
White blood cell (/μl)	4170 (3300–5100)
Hemoglobin (g/dl)	13.2 (12.1–14.2)
Platelets (x10^4^/μl)	11.4 (8.2–15.5)
Total bilirubin (mg/dl)	0.73 (0.67–1.00)
AST (IU/l)	52 (37–74)
ALT (IU/l)	48 (31–69)
γGTP (IU/l)	32 (24–54)
Creatinine (mg/dl)	0.71 (0.59–0.85)
Albumin (g/dl)	3.8 (3.6–4.2)
History of HCC treatment: No/Yes	259 / 63
Comorbidity of diabetes mellitus: No/Yes	249 / 73
NS5A-L31M/V: negative / weak positive / positive	270 / 22 / 5
NS5A-Y93H: negative / weak positive / positive	235 / 57 / 10

IFN, interferon; RBV, ribavirin; Peg-IFN, Peginterferon; TVR, telaprevir; SMV, simeprevir; AST, aspartate transaminase; ALT, alanine aminotransferase; γGTP, γ-glutamyl transpeptidase; HCC, hepatocellular carcinoma

### Treatment outcomes and factors associated with SVR

Among the 322 patients, 13 patients (4.0%) discontinued ASV/DCV therapy due to adverse effects during the treatment at 2 to 23 weeks after the start of treatment. The causes of treatment discontinuation were elevation of alanine aminotransferase (ALT) levels (7 patients), eruption (3 patients), high fever, sore throat and headache (one patient each). However, all patients except one who discontinued due to eruption at 3 weeks (58 years, male) achieved SVR. The overall SVR rate was 93.8% (302/322). Factors associated with SVR are shown in **Table 2**. The previous history of SMV-based triple therapy and NS5A-L31M/V and/or Y93H positive by Invader assay were independently associated with SVR by multivariate analysis. According to NS5A substitutions by Invader assay, the SVR rates for patients with NS5A-Y93H and/or L31M/V positivity and non-positivity were 57.1% and 95.8%, respectively. The SVR rate for patients with a previous history of SMV-based triple therapy was 70% (7/10).

**Table 2:** Factors associated with SVR.

Factor	Category	Univariate analysis			Multivariate analysis		
		Odds ratio	95% CI	P value	Odds ratio	95% CI	P value
Age (y.o)	≤71 vs. 71<	2.771	0.982–7.815	0.054			
Sex	Male vs. female	1.254	0.507–3.101	0.625			
Body mass index (kg/m^2^)	≤22.6 vs. 22.6<	1.104	0.436–2.795	0.835			
History of IFN therapy	No vs. Yes	0.524	0.204–1.351	0.181			
History of SMV/Peg-IFN/RBV	No vs. Yes	0.136	0.032–0.572	0.007	0.115	0.020–0.640	0.014
Liver cirrhosis	No vs. Yes	0.51	0.205–1.269	0.148			
HCV-RNA (log IU/ml)	≤6.1 vs.6.1<	0.546	0.217–1.374	0.199			
Liver histology: Fibrosis	F0-2 vs. F3-4	0.867	0.295–2.549	0.795			
Hemoglobin (g/dl)	≤13.2 vs. 13.2<	1.07	0.431–2.658	0.883			
Platelets (x10^4^/μl)	≤11.4 vs. 11.4<	1.932	0.750–4.977	0.172			
Total bilirubin (mg/dl)	≤0.73 vs. 0.73<	0.987	0.399–2.440	0.977			
ALT (IU/l)	≤48 vs. 48<	0.924	0.374–2.283	0.863			
Albumin (g/dl)	≤3.8 vs. 3.8<	0.87	0.344–2.203	0.769			
Creatinine (mg/dl)	≤0.71 vs. 0.71<	0.764	0.308–1.900	0.564			
Comorbidity of DM	No vs. Yes	1.185	0.383–3.660	0.769			
AFP (ng/ml)	≤8 vs. 8<	0.942	0.381–2.329	0.897			
NS5A-L31 and/or Y93 substitutions	non-positive* vs. positive	0.059	0.018–0.197	<0.001	0.051	0.015–0.175	<0.001

*Negative and weakly positive results for NS5A-L31 and/or Y3 substitutions are considered non-positive results.

SVR, sustained virological response; IFN, interferon; SMV, simeprevir; Peg-IFN, Peg-interferon; RBV, ribavirin; ALT, alanine aminotransferase; DM, diabetes mellitus; AFP, α-fetoprotein

### Profile of non-SVR patients

Excluding a discontinued patient due to adverse effects, 19 patients failed to achieve SVR. Among them, 3 patients had a previous history of SMV-based triple therapy and experienced breakthrough during treatment, and 2 patients were identified as having genotype 1a infection and experienced nonresponse. The outcome of the other 14 non-SVR patients was as follows: 7 patients relapsed; 5 patients had breakthrough; and 2 patients had no response. After virologic failure, results were obtained for 11 out of 14 patients for deep sequencing, and 10 patients had linked L31M/V-Y93H double substitution at high frequency (93.0 ~ 99.4%), as described previously [12]. One patient without L31 and Y93 substitution had NS5A-P32 deletion (99.7%) after virologic failure.

### The pre-existing linked NS5A-L31-Y93 double substitution in the case-control study

We performed a case-control study to compare the prediction of treatment effect by a substitution at either position before treatment by conventional Invader methods or double substitutions at these sites by deep sequencing. The non-SVR group consisted of 14 non-SVR patients, excluding patients with previous SMV exposure or genotype 1a infection from 19 treatment failures. In the SVR group, 42 patients were randomly selected from the present cohort as the control group. Before random selection, 20 patients who discontinued treatment, 11 patients with a history of previous TVR and/or SMV exposure and 19 patients who did not have data on NS5A substitution from the Invader method were excluded from the SVR group. The patient backgrounds of the non-SVR and SVR groups in the case-control study are shown in **S1 Table.** There was no difference in each variable between the non-SVR group and the SVR group. The results of the Invader assay for NS5A-L31M/V and NS5A-Y93H at baseline and the frequency of the L31M/V-Y93 single substituted strain, L31-Y93H single substituted strain and L31M/V-Y93H double substituted strain analyzed by deep sequencing are presented in **Table 3**. The sequence results of one patient in the SVR group were not determined because of unsuccessful PCR amplification. The frequency of substitutions in both positions by the conventional method was very low, with 1 in 14 non-responders and 0 in 42 randomly selected responder patients. In the non-SVR group, linked L31M/V-Y93H double substituted strains were detected in 8 patients, ranging from 0.1 to 38.9% of the population. Among them, the population of 6 patients was less than 1%. On the other hand, only one patient had double substituted strains at a frequency of 0.1% in the SVR group. In the conventional method, substitutions were detected at any position in 6/14 non-responders and 2/42 responders, and there was a clear difference between the two groups. The difference was also clear in the deep sequencing method, with substitutions in 11/14 non-responders and 8/42 responders. Interestingly, in the deep sequencing method, the single substitution of L31 was in 6/14 non-responders and 7/42 responders, and the difference was small, but single substitutions of Y93 or double substitutions were 7/14 and 8/14, respectively, in non-responders, and 1/42 and 1/42 in responders, and there was a marked difference between the two groups.

**Table 3:** The results of pre-existing NS5A L31M/V and/or Y93H substitution analyzed by Invader methods and deep sequencing for non-SVR patients and randomly selected SVR patients.

Case	Effect	Invader	Invader	Deep	Deep	Deep	Case	Effect	Invader	Invader	Deep	Deep	Deep
		L31M/V	Y93H	L31M/V-Y93	L31-Y93H	L31M/V-Y93H			L31M/V	Y93H	L31M/V-Y93	L31-Y93H	L31M/V-Y93H
1	Non-SVR	M/V (+)	H (+)	M 57.0%	H 0.8%	M-H 38.9%	29	SVR	(-)	(-)	(-)	(-)	(-)
2	Non-SVR	(-)	H (+)	V 3.2%	H 57.6%	V-H 12.9%	30	SVR	(-)	(-)	(-)	(-)	(-)
3	Non-SVR	(-)	H (+)	(-)	H 96.2%	V-H 0.3%	31	SVR	(-)	(-)	(-)	(-)	(-)
4	Non-SVR	(-)	(-)	(-)	H 59.8%	M-H 0.3%	32	SVR	(-)	(-)	(-)	(-)	(-)
5	Non-SVR	M/V (+)	(-)	M 98.4%	(-)	M-H 0.2%	33	SVR	(-)	(-)	(-)	(-)	(-)
6	Non-SVR	(-)	(-)	(-)	(-)	M-H 0.2%	34	SVR	(-)	(-)	(-)	(-)	(-)
7	Non-SVR	(-)	H (+)	(-)	H 99.2%	V-H 0.1%	35	SVR	(-)	(-)	(-)	(-)	(-)
8	Non-SVR	(-)	(-)	M 0.4%	H 0.3%	M-H 0.1%	36	SVR	(-)	(-)	(-)	(-)	(-)
9	Non-SVR	(-)	(-)	(-)	H 3.0%	(-)	37	SVR	(-)	(-)	(-)	(-)	(-)
10	Non-SVR	(-)	(-)	M 0.7%	(-)	(-)	38	SVR	(-)	(-)	(-)	(-)	(-)
11	Non-SVR	M (+)	N.D.	M 2.6%	(-)	(-)	39	SVR	(-)	(-)	(-)	(-)	(-)
12	Non-SVR	(-)	(-)	(-)	(-)	(-)	40	SVR	(-)	(-)	(-)	(-)	(-)
13	Non-SVR	(-)	(-)	(-)	(-)	(-)	41	SVR	(-)	(-)	(-)	(-)	(-)
14	Non-SVR	(-)	(-)	(-)	(-)	(-)	42	SVR	(-)	(-)	(-)	(-)	(-)
15	SVR	V (+)	(-)	V 98.0%	(-)	V-H 0.2%	43	SVR	(-)	(-)	(-)	(-)	(-)
16	SVR	(-)	H (+)	(-)	H 9.5%	(-)	44	SVR	(-)	(-)	(-)	(-)	(-)
17	SVR	(-)	(-)	M 1.1%	(-)	(-)	45	SVR	(-)	(-)	(-)	(-)	(-)
18	SVR	(-)	(-)	M 0.9%	(-)	(-)	46	SVR	(-)	(-)	(-)	(-)	(-)
19	SVR	(-)	(-)	M 0.8%	(-)	(-)	47	SVR	(-)	(-)	(-)	(-)	(-)
20	SVR	(-)	(-)	M 0.5%	(-)	(-)	48	SVR	(-)	(-)	(-)	(-)	(-)
21	SVR	(-)	(-)	M 0.4%	(-)	(-)	49	SVR	(-)	(-)	(-)	(-)	(-)
22	SVR	(-)	(-)	M 0.2%	(-)	(-)	50	SVR	(-)	(-)	(-)	(-)	(-)
23	SVR	(-)	(-)	(-)	(-)	(-)	51	SVR	(-)	(-)	(-)	(-)	(-)
24	SVR	(-)	(-)	(-)	(-)	(-)	52	SVR	(-)	(-)	(-)	(-)	(-)
25	SVR	(-)	(-)	(-)	(-)	(-)	53	SVR	(-)	(-)	(-)	(-)	(-)
26	SVR	(-)	(-)	(-)	(-)	(-)	54	SVR	(-)	(-)	(-)	(-)	(-)
27	SVR	(-)	(-)	(-)	(-)	(-)	55	SVR	(-)	(-)	(-)	(-)	(-)
28	SVR	(-)	(-)	(-)	(-)	(-)	56	SVR	(-)	(-)	N.D.	N.D.	N.D.

SVR, sustained virological response; N.D., not determined, (-), negative or non-positive, (+), positive

### A comparison of the predictive capability for the treatment effect according to resistance tests

**Fig 2:**
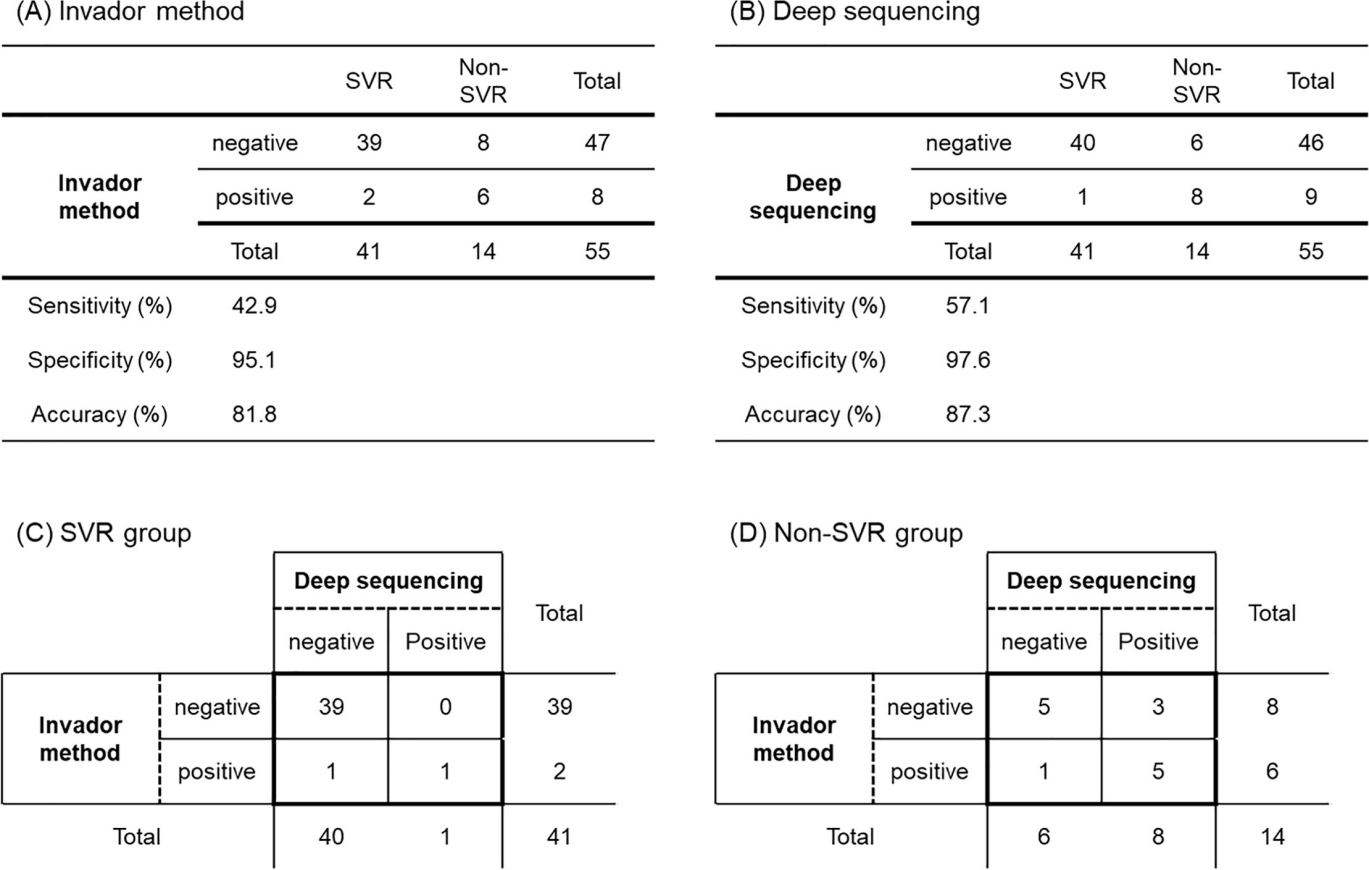
Cross tabulations of numbers according to the Invader method and deep sequencing.

Although the difference was not statistically significant, the sensitivity for the probability of virologic failure from double substitution revealed by deep sequencing was higher than that from substitution at either position by the Invader method (57.1% [8/14] vs 42.9% [6/14], p = 0.625) (**Fig 2A and 2B**). In the SVR group, as many as 39 patients out of 41 patients (one patient without successful PCR amplification was excluded because the patient was not included in either the negative or positive group) were negative for both resistance tests (**Fig 2C**). The specificity for the probability of virologic failure from both resistantce tests was high (97.6% [40/41] vs 95.1% [39/41], p = 1.000). Cross-tabulations of numbers according to the Invader method and deep sequencing are shown in **Fig 2C and 2D.** In the non-SVR group, the results of 10 patients matched each other (five patients: positive, five patients: negative). However, 3 patients who were negative for NS5A-Y93H and NS5A-L31M/V by the Invader method revealed positive double substituted strains by deep sequencing. The real non-SVR rate for patients without previous SMV exposure was 5.45% (17/312). The estimated SVR rate for patients with positive for double substitution by deep sequencing calculated by sensitivity, specificity and entire SVR rate [100%-(5.45%×57.1%)/(5.45%×57.1%+(100%-5.45%)×(100%-97.6%)) = 42.2%] was lower than the real SVR rate for patients with positive for substitution at either position by the Invader method (42.2% vs 57.1%).

## Discussion

NS5A L31M/V and Y93H substitutions appear at virologic failure for DAA therapy with NS5A inhibitors. Strong resistance to DAA therapy and long-term sustainability of NS5A L31M/V and Y93H substitution affects DAA treatment choice. With conventional sequencing, approximately 1% of DAA-naive patients are positive for both L31M/V and Y93H substitutions. However, the frequency of linked NS5A L31M/V-Y93H double substitution is unclear. Recently, the development of deep sequencing technology enabled us to detect linked L31M/V-Y93H double substitutions. To analyze linked L31-Y93 substitutions in a single clone, we designed a primer set between which both L31 and Y93 were located and applied a fusion method. As a result, we established a method to detect linked NS5A L31M/V -Y93H double substitution.

In this study, which was limited to genotype 1b infection, we analyzed the usefulness of deep sequencing analysis for detection of pre-existing NS5A L31M/V-Y93H double substitution to predict therapeutic effects compared with the Invader method. We performed a case-control study to compare the predictive ability of the DAA treatment effect using deep sequencing and the conventional Invader assay in the SVR group and non-SVR group. The positive rate of double substituted strains was 57.1% (8/14) in the non-SVR group and only 2.4% in the SVR group. Deep sequence analysis was superior in sensitivity for the prediction of non-SVR compared with the Invader method and was slightly better in specificity than the Invader method, although both methods showed high specificity. The accuracy of Invader methods for predicting therapeutic effects is limited compared with that of deep sequencing. We also detected a D168 mutation, which was a major mutation of the NS3 region, but only one non-responder without NS5A mutations had the D168 mutation before treatment. The presumed SVR rate of double substituted strains by deep sequencing was lower than that by the Invader method. Whereas double substituted strains were 1% less frequent in 6 cases of 8 cases with double substituted strains in the non-SVR group, Kinugasa H reported that minor clones of NS5A L31M/V or Y93H substitution were not associated with treatment effect [20]. However, we already reported that two cases had less than 1% minor clones, which was the origin of non-SVR clones by phylogenetic tree analysis [12], which may mean that minor clones of the L31M/V-Y93H double substitution can relate to virologic failure for ASV/DCV due to its strong drug resistance.

In this study, we examined baseline RASs by deep sequencing of patients who started ASV/DCV treatment. Although various regimens are available for GT1b hepatitis C virus infection, DCV and ASV combination therapy is still used and is one of the initial treatments for chronic hepatitis C or compensated cirrhosis patients with HCV genotype 1b infection in the Asian Pacific Association for the Study of the Liver (APASL) clinical practice recommendation [21]and the 2017 KASL Clinical Practice Guidelines for Management of Hepatitis C: Treatment of Chronic Hepatitis C [22]. Furthermore, testing for NS5A RASs is recommended prior to DCV and ASV combination therapy in these guidelines. Since patients who had been determined to have NS5A RASs by the Invader method tended to avoid treatment, the frequency of double substitution shown in this study may be lower than that of the general hepatitis C population. Of course, we must consider the cost of deep sequencing methods and alternative regimens that are less susceptible to RASs. In fact, the numbers of patients who were positive for NS5A-L31M/V and NS5A-Y93H by the Invader assay among 630 consecutive patients with chronic HCV genotype 1 were 27 (4%) and 75 (12%), respectively. However, the important point is that double mutations in tandem may be detected even in patients where mutations are not detected by conventional methods, or only one of them is detected, which are related to the therapeutic effect.

In conclusion, in genotype 1b naive patients, cases where mutations are detected in both L31 and Y93 positions by the conventional method are extremely rare, but using the deep sequencing method, mutations have been detected in tandem in these positions in several patients, who may be more resistant to DAA treatments containing NS5A inhibitors. Pre-existing double mutation substitution, which is involved in ASV/DCV virologic failure, is more accurately detected by deep sequencing. The deep sequencing is highly sensitive for low prevalence mutations that limit the utility of the ASV/DCV treatment option in these patients.

## Supporting information

S1 Table Baseline characteristics of patients in non-SVR group and randomly selected SVR group. (XLSX)
